# Shaping the Future of Shared Services Centers: Insights from a Delphi Study About SSC Transformation Towards 2030

**DOI:** 10.1007/s13132-022-01072-0

**Published:** 2022-10-10

**Authors:** Cicero Ferreira, Marijn Janssen

**Affiliations:** 1 University of Liverpool, Liverpool, England; 2 Delft University of Technology, Delft, Netherlands

**Keywords:** Shared service centers, Shared services, Organizational transformation, Systems theory, Disruption by technologies, Dynamic capability, Digital transformation, Delphi, Foresight

## Abstract

In recent years, shared services centers (SSCs) have been central to organizational transformation strategies of many large firms and governments to reduce costs, improve service quality, and innovate services. SSCs are undergoing significant transformations due to the advancement of technologies. To better understand the technologies’ impact on SSCs in 2030, a Delphi panel with over 30 experts, executives, and researchers was conducted in the first half of 2020. Panelists pointed out six recent technologies potentially impacting SSCs by 2030: AI/ML, internet/package-based automation, BPMS/RPA, business analytics, blockchain, and cloud computing. The experts expect that these technologies will cause a transformation in the SSC business model and disruptive changes in SSC employees’ and managers’ profiles. Managers cannot merely continue their regular efforts and should refocus from automating repetitive functions towards intensifying the use of technology in value-adding services. The outcomes should help public and private sector managers to be ahead of change and prepare their organizations for the future of SSCs.

## Introduction

Over the years, countless large public and private organizations have embraced shared service centers (SSCs) to reduce cost and increase service levels and innovation capabilities at the same time (Lakshmi et al., 2020). SSCs are organizational arrangements in which activities are unbundled and concentrated in a separate semi-autonomous unit (Bergeron, 2002; Janssen et al., 2007). SSC’s basic premise is that services provided by one local department can be delivered to other departments with relatively few efforts (Bergeron, 2002). The introduction of SSCs requires a transformation in the way organizations are organized (Boglind et al., 2011; Weerakkody et al., 2011). Since the 1990s, SSCs have invested in technology, mainly focusing on automating transactional activities and services to increase productivity and reduce operational costs (Bergeron, 2002). However, the advance of technology in the twenty-first century has accelerated that even the most specialized and strategic activities might be impacted by recent advances, such as artificial intelligence (AI).

SSCs are undergoing significant transformations due to the advancement of technologies (Lakshmi et al., 2019; Suri et al., 2017; Willcocks et al., 2017). The research presented in this article aims to understand the impact that new technologies will have on SSCs in the next 10 years. The motivation of the study is to help managers, in both the public and private sectors, to be ahead of change and prepare their organizations for the future of SSCs. To select critical themes, identify emergent issues during data collection and analysis, and guide the research process (Saunders et al., 2016), the study considered the key technologies used by SSCs in 2020 with the potential to impact their future. SSCs’ future is difficult or even impossible to predict, as SSCs are complex and multifaceted arrangements (Schulman et al., 1999). Beyond short-term forecasts, the future can sometimes be contradictory or paradoxical (Bouwman & van der Duin, 2003; Handy, 2011). Therefore, we opted for a Delphi method with a variety of persons having different opinions and visions. Panelists pointed out six recent technologies potentially impacting SSCs by 2030, as shown in Table 1.

**Table 1:** Technologies potentially disrupting SSCs by 2030

#	Technology
1	Artificial intelligence/machine learning. Learning and improving from data without being programmed (Iandolo et al., 2021; Kashyap, 2018; Simon, 2019).
2	Internet/package-based automation. Internet—The global computer network system enabling faster and immediate information availability and transaction (Xue et al., 2022). Package-based automation—The use of a ready-made solution to automate an organizational function (e.g., HR system) or an entire business (e.g., core banking system, insurance system).
3	Business process management systems/robotic process automation. BPMS employs explicit process representations to coordinate the enactment of business processes (Weske, 2007). RPA is the automation of repeatable, high-volume tasks (emulating human action) (Accenture, 2020).
4	Business analytics. The use of data to allow more informed business decisions and produce actionable insights (Holsapple et al., 2014; Iandolo et al., 2021; Monino, 2021).
5	Blockchain. Technology designed to ensure data security and having trusted transactions (Osmani et al., 2020; Roberts & Karras, 2019).
6	Cloud computing. On-demand access and use of applications over the internet, enabling fast and easy scaling (Kashyap, 2018).

The panelists expect that these technologies will cause radical transformations in the business model and SSC employees’ and managers’ profiles. A business model defines the logic of how value is created, marketed, delivered, and captured by an organization (Bašić, 2022; Díaz-Díaz et al., 2017; Osterwalder et al., 2005; Wilson, 1992). We define the SSC business model in the “SSC Business Models Transformation” section.

The nature of the study is exploratory, and the paper is structured as follows. In the next section, the “Theoretical Foundation”, we discuss the theoretical basis of the study. The following section, the “Research Approach,” clarifies the paradigm that guided the study and the methodology used for sample definition, data collection, and analysis. Next, in the “Findings” and “Discussion of the Impact on SSCs” sections, we present the research’s main findings and discuss how new technologies should impact SSCs and their professionals in the next 10 years. In the last section, the “Conclusions,” the main findings and their significance are highlighted. Recommendations are also made for future research. Appendices 1 and 2 present the questionnaires used in the first and second Delphi rounds.

## Theoretical Foundation

The study aims to identify the impact of emerging technologies on SSCs over the next 10 years—what changes should occur in the SSCs due to the adoption and use of new technologies? Foresight studies have broad theoretical and methodological support (Giaoutzi & Sapio, 2013). The study’s theoretical basis is formed by elements of the systems theory, contingency theory, and dynamic capabilities. The reasons for choosing these theories are explained in the following paragraphs. This theoretical background underpins the problem’s conception, the research questions, the research design, and its conceptual lenses to examine the data collected.

Often, SSCs operate within a broad scope. Systems theory views the organization as an integrated whole, with interacting parts, inserted in an environment with which it continuously interacts (Chiavenato, 2014; Haile & Altmann, 2016; Johnson et al., 1964). From systems theory, we used three concepts in this study. The first, the concept of open systems, explores organizations’ interaction with their external environment, the exchange of information, and its adaptation to continuing in balance (Johnson et al., 1964). In the case of SSCs in this study, this concept helps us, for example, to understand that, as open systems, SSCs compete with other systems—other potential service suppliers—and need to remain competitive, transforming themselves to continue adding value to their clients over time. It also emphasizes the need to adapt to changes in the environment. The second concept, models of organization*—*e.g., Schein model and Katz and Kahn Model (Chiavenato, 2014)—gives us the vision that an organization is a set of mutually dependent subsystems, and changing one affects the behavior of other subsystems. This perspective enlightens that the impact of new technologies on SSCs transcends their boundaries and should be evaluated considering the whole company. As the last of the three concepts, the processes*—*a set of interdependent and interacting elements (with time-ordered sequences of tasks) to achieve a goal or purpose—help us reflect on the execution of the organization’s tasks. In this study, this vision allows, for example, to reflect on the consequences of task automation in the provision of services by SSCs.

SSCs are contingent (dependent) upon their external and internal context (Chiavenato, 2014). Furthermore, contingency theory suggests that any organization, by extension, the SSC, is dependent on the context and emphasizes that the environment’s conditions cause transformations in organizations (Donaldson, 2001). This emphasizes technology as an element of the environment that is changing. As organizations live in a changing world, their organizational model must be characterized by flexibility and adaptability to the environment and technology. To survive and remain competitive, each SSC and any organization need to incorporate technology that comes from the environment, which determines their organizational design and task environment. Although this theory has its roots in relativism, and this research has its bases in constructionism, we benefit from a dialogue between these viewpoints that help us to study the role of technology in the context of SSCs.

In the future of SSC, new organizational capabilities will likely be needed. Dynamic capabilities reflect the firm’s ability to integrate, build, and reconfigure internal and external competencies to address rapidly changing environments (Ferreira et al., 2021; Teece, 2009). And dynamic capabilities theory (Eisenhardt & Martin, 2000; Teece, 2007; Teece & Pisano, 1994) provides a conceptualization and model for organizations to do that. In this study, the theory helps identify the dimensions that should be impacted by new technologies (e.g., competencies, processes, learning, adaptability, decision-making, and organizational skills (Tondolo & Bitencourt, 2014)) and how SSCs might evolve the need for those capabilities. Dynamic capabilities are particularly relevant in technological change because the SSCs will need to identify and respond to new opportunities (Janssen & Joha, 2006a), mainly in the context of technological changes, which requires a reconfiguring of their capabilities. Table 2 summarizes how systems theory, contingency theory, and dynamic capabilities help conceptualize SSCs and address the surrounding issues.

**Table 2:** Main theories supporting this study

Theories	Concepts useful to understand SSCs and prepare them for the future	Elements of SSCs impacted by the concept	Comments	References
Systems theory	Open systems	• Interaction with the external environment • Exchange of information • SSC adaptation to continuing in balance	SSCs compete with other potential service suppliers and need to remain competitive, transforming themselves to adapt to changes in the environment	Johnson et al. (1964), Schein Model, and Katz and Kahn Model (Chiavenato, 2014)
	Models of organization	• SSC is one of the mutually dependent subsystems	The impact of new technologies on SSCs should be evaluated considering the whole company	
	Processes	• SSC as a set of interdependent and interacting elements to achieve a goal or purpose	This vision allows us to reflect on the consequences of task automation in the provision of services by SSCs	
Contingency theory	Organizations are contingent (dependent) on the context	• Environment conditions cause transformation in organizations (e.g., SSC)	To survive and remain competitive, each SSC needs to incorporate technology that comes from the environment, which determines their organizational design and task environment	Donaldson (2001)
		• Technology as an element of the environment that is changing		
		• The organizational model must be flexible and adaptable		
Dynamic capabilities theory	The firm’s ability to integrate, build, and reconfigure internal and external competencies	• Technological changes require a reconfiguring of organization capabilities	The theory helps identify the dimensions that should be impacted by new technologies (e.g., competencies, processes, learning, adaptability, decision-making, and organizational skills)	Eisenhardt and Martin (2000), Teece (2007), Teece and Pisano (1994), Tondolo and Bitencourt (2014)

The scientific administration emphasized the homo economicus, while the school of human relations emphasized the social man (Chiavenato, 2014; Longo, 2007). Structuralism brought out the organizational man, while behavioral theory highlighted the administrative man. In recent times, the academy has turned its attention to the homo digitalis, whose transactions with their environment are predominantly carried out through the computer and the internet (Chiavenato, 2014; Kotler et al., 2020). The intensive use of technology by the homo digitalis in their daily lives and organizations reaches levels never imagined, even in shared service centers.

## Research Approach

To understand which new technologies should gain traction and be relevant to SSCs in the future and estimate their impact, our research method aims to capture highly specialized IT and SSC knowledge. This high level of expertise is captured using opinions from experts, who are up to date with the latest knowledge and trends. The research should also give managers insights to anticipate changes and adjust SSCs gradually to the changes to come. The study’s aim calls for qualitative research, as it makes it possible to understand the perspective of study participants and interact with them. These needs and the context of the research led us to adopt as research strategy the Delphi method, whose primary use is in forecasting and discussions about the future (Linstone & Turoff, 1975).

Therefore, the study follows the social constructionist paradigm (Easterby-Smith et al., 2008), which asserts that reality depends on the observer, and knowledge is socially constructed (Zhao, 2022); thus, it should be given special attention to the language and conversations. As the research strategy, we used the Delphi method (Linstone & Turoff, 1975; Okoli & Pawlowski, 2004; Wright et al., 2000). Such an approach facilitates the asynchronous interaction of experts in a panel to anticipate opportunities and threats in discussions about the future and define the appropriate actions to be taken today (Giaoutzi & Sapio, 2013). Compared to other methods, such as traditional survey and scenario planning, the Delphi method enables individual and collective reflection on the issues addressed, without the disadvantages of in-person meetings, and provides a synergy of ideas and perceptions among experts. The Delphi approach also increases the process knowledge through the improvement and evolution of the questions asked. It similarly enhances the use of the expertise and experience of specialists.

### Delphi Method

The Delphi method structures communication within a group to deal with a complex problem (Linstone & Turoff, 1975; Wright et al., 2000). Complex problems have the following characteristics (Funke, 2010): (a) the elements involved in the solution are numerous. For instance, organizational elements (e.g., people, culture, technology, clients, competitiveness), impacted dimensions (e.g., efficiency, services, innovation), and the level of impact (e.g., low, medium, high); (b) many elements are interconnected; (c) they change over time (dynamics). These three characteristics are present in the analysis of the impact of new technologies on the future of SSCs.

The Delphi method is based on three assumptions: (i) anonymity of the respondents, (ii) statistical representation of the responses, and (iii) the return of group responses for reassessment in subsequent rounds. The Delphi method dynamics advance the discussion into a consensus, representing a consolidation of the expert’s intuitive judgment on future events and trends (Linstone & Turoff, 1975; Wright et al., 2000). Such a judgment is made possible by the structured use of knowledge, experience, and creativity of the expert panel and considers the principle that collective judgment, adequately organized, is better than individual opinion. The Delphi method is especially recommended when quantitative data are not available or cannot be safely projected for the future, given the expected structural changes in the determining factors of future trends (Linstone & Turoff, 1975; Wright et al., 2000). The size of the expert panel may vary depending on the purposes of the study. The authors suggested not having less than 7 to 12 people for a Delphi panel (Baldwin & Trinkle, 2011).

Conducting the Delphi method is relatively simple; questionnaires are submitted in several rounds to a group of experts, preserving the answers’ anonymity. In the first round, the experts receive the questionnaire—or a link for online access—prepared by the coordination team and answer the closed-ended or open-ended questions individually. The answers to open-ended questions receive content analysis treatment to enable their coding and structured analysis. The answers to the closed-ended questions are tabulated, given a simple statistical treatment, and the results are returned to the participants in the next round. In each round, the previous round’s responses consolidated are submitted to the panel, and the panelists reevaluate their reactions considering the justifications of the other respondents. The process can be repeated in successive rounds until the divergence of views is reduced to a satisfactory level. The response from the last round is considered the panel’s forecast.

Regarding the time horizon of the forecasts, although it varies from sector to sector, a prospective study may focus on the short term (from 1 to 3 years), medium term (from 4 to 10 years), or long term (usually above 10 years) (Brier, 2005). In this study, we adopted a 10-year perspective so that SSC managers in the field can reference their SSC adequacy actions and the appropriate time to do so. The time horizon might be influenced by exogenous developments, such as the acceleration of technology development due to the COVID-19 pandemic, as explored in the “Conclusions” section.

### Sample Selection

The sample was aimed at gaining a diversity of perspectives and views. To form the Delphi panel with IT and SSCs specialists, we counted on executives and experts from SSCs, academia, and consultancy firms such as IBM and Deloitte, from many parts of the world, like North and South America and Europe. Initially, we used a “sample for convenience” of our professional contacts. We then used the networking sampling approach (Dillman et al., 2014), interacting with three potential sources: (a) SSC LinkedIn groups—Finance Shared Services Best Practice Network, with 817 members, and Shared Services Best Practice Network, with 5706 members; (b) international SSC conference contacts in the USA and the UK (103 people); and (c) ABSC—Associação Brasileira de Serviços Compartilhados, with 325 members. In Delphi studies like this, the panelists’ quality determines the quality of the research results, so we restricted the invitations to experts and executive-level professionals to the research sampling frame. In the first half of 2020, we sent about 500 invites to potential Delphi panelists, resulting in 60 confirmations. Due to our study’s nature, we opted for having a mix of technical and managerial functions of the panel members. Moreover, we invited only experienced professionals (at least 10 years of experience), resulting in the profile shown in Table 3.

**Table 3:** Background of the panel members

Background	# of panelists	Occupation
IT/SSC	18	• 4 CIOs/directors • 4 IT experts • 8 senior managers/consultants • 1 university technology researcher • 1 university SSC researcher
SSC	41	• 31 directors • 7 senior managers/consultants • 3 SSC experts
Delphi method	1	• 1 university Delphi researcher
Total	60	

### Data Collection and Analysis

The data collection took place in the first half of 2020 in two rounds of questionnaires whose online links were e-mailed to Delphi panel experts. In the first Delphi round, there were 33 participants, and in the second Delphi round, 32 participants, keeping the proportion of about 30% of IT background panelists. Twenty-nine of the respondents (90%) participated in both rounds. The abstention of 47% concerning the 60 initially confirmed participants is within the expected range, 30 to 50%, according to Wright et al. (2000).

A questionnaire was submitted in the first round to understand the impact of new technologies on SSCs over the next 10 years. The questionnaire contained seven open-ended questions: (1) Which five technologies will have the most significant impact on SSCs in the next 10 years? (2) How will each of these five technologies impact the future of SSCs? (3) Do you expect that the need for SSCs (company-internal and outsourced) by customers will decline or increase due to those technologies? (4) How will the SSC internal business model be transformed? (5) Will the nature of services and activities provided by SSCs change over the next 10 years? Will new technologies make SSCs more strategic for organizations? (6) How will new technologies affect workers’ and SSC leaders’ profiles? (7) What is the primary value added today by the SSCs, and how will this change 10 years from now? A structured content analysis was performed to analyze and interpret the answers.

With the support of QDA Miner Lite software (Adu, 2019), we conducted content analysis to evaluate the answers to the open-ended questions, which is a systematic way to arrange, explain, and analyze the content of texts and surveys. The content analysis was performed in three steps using a technique adapted from the work of Bardin (1977) and Creswell (2007). The first step, called “pre-analysis,” consisted of reading the answers, providing the information needed for the next stage of the categorization. The second step, “exploration’ of the material, comprised a more thorough analysis with text excerpts and categorizations attempting to isolate, group, and describe the concepts (codes) present in the texts analyzed. Finally, the third step, “interpretation,” clarified the stated or latent original content of the analyzed data. Creating categories at this point takes into account Bardin’s five principles (Bardin, 1977): mutual exclusion, between categories; homogeneity, within categories; relevance, or no distortion, of the transmitted message; fertility for inferences; and objectivity or comprehension and clarity.

After coding and interpreting the first questionnaire’s answers, the second questionnaire was designed with closed-ended questions using a Likert scale, multiple choice, and selection boxes (Appendix 2). The second questionnaire’s purpose was to verify the impact of the technologies most cited by respondents in the first round on SSCs. To identify what skills will be needed by SSC professionals of the future, we used the skills model proposed by Van Laar et al. (2017) for employee skills and the skills model proposed by Sousa and Rocha (2019) for management skills.

As recommended for social research, participants were informed in writing of the voluntary and anonymous nature of their participation in the study and that they would be able to interrupt their activities at any time if they so wished (Easterby-Smith et al., 2008). They were also informed that their data in the questionnaires would be encrypted and kept anonymous under secure access.

In the next section, we organize the presentation of the findings into three blocks. In the first block, we address the technologies highlighted by the panelists as those that will most impact the SSCs. In the second, we underline the profile required of SSC professionals in the future to succeed in a very different working context from today’s, both in service execution and in SSC leadership. In the final block, we present the panelists’ vision of what should be transformed in the SSC business model and discuss the value added by SSCs to business and in which direction it probably will evolve.

## Findings

In the following subsections, we present the research findings and analyze their significance from a theoretical and practical perspective.

### Impact of New Technologies in SSCs over the Next 10 Years

In the first Delphi round, panelists were asked what technologies would significantly impact SSCs over the next decade. Six technologies were the most cited:

- AI/ML is expected to have the most significant impact on SSCs by 2030 for 78% of the respondents. At present, with a few exceptions, SSCs are just only exploring this technology or using AI/ML quite timidly (SSON, 2020) in simple tasks, such as chatbots. Shortly, this technology is expected to revolutionize the SSCs, with decision support and forward-looking data analysis.
- Internet/package-based automation will also have a significant impact, according to 62% of the respondents. Today, the automation is limited to software packages (e.g., ERPs, PSA), while by 2030, SSCs will expand the use of other technologies, such as BPMS/RPA, to integrate their solutions.
- BPMS/RPA was also mentioned by 62% of the respondents as high-impact technologies for SSCs. The evolution of these technologies is enabling viable intelligent process automation (Lakshmi et al., 2020; SSON, 2020), which combines redesign, automation, end-to-end process management, and continuous improvement using AI/ML and RPA.
- Business analytics was mentioned by 37% of the responses. Nowadays, the most common use of BA is still restricted to traditional BI tools for analyses. By 2030, SSCs should use algorithms to get models to support predictive analytics, neural networks, and complex event processing. This will allow them to have a more consultative role to support the business units.
- Blockchain was also mentioned by 37% of the respondents. Blockchain would enable automatic transactions between parties and the safe and secure storage of data. SSC could facilitate and maintain the blockchain to enable users to transact.
- Cloud computing got 34% of the responses. SSCs could help users migrate to the cloud and make agreements, while public sector SSCs could operate their own cloud within the national boundaries. In this way, ensuring that data is only stored with the jurisdiction of the country.

**Fig. 1:**
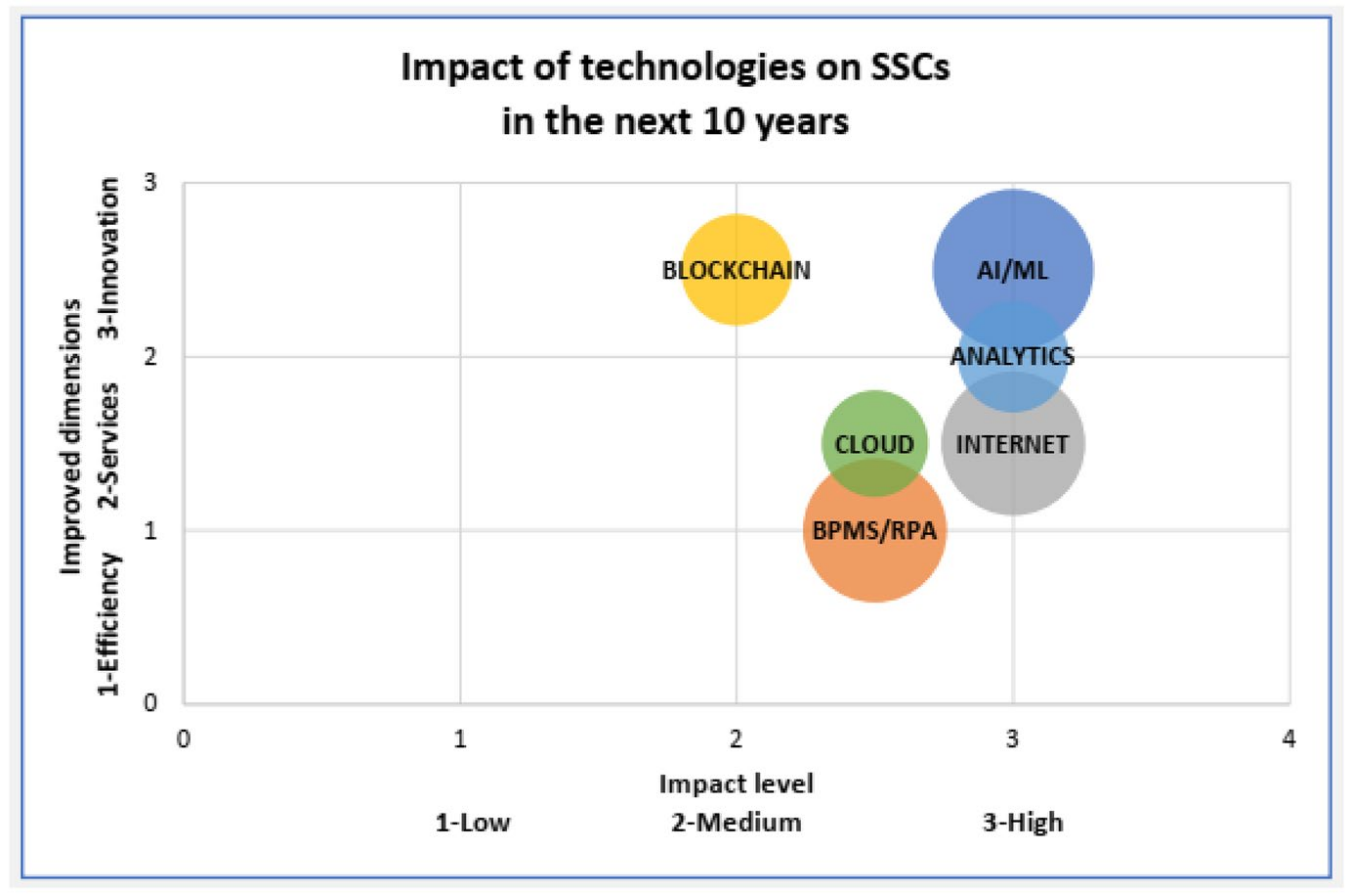
Impact of disruptive technologies on SSCs

In the second Delphi round, the panelists qualified the level and type of impact these technologies will have on SSCs. The convergence of views among participants was faster than expected (only two rounds), although the experts were selected for their diversity in opinions. This can be explained by the relatively well-defined boundaries and concept of SSCs. The scatter plot of Fig. 1 below summarizes the technologies’ impact, including whether these technologies will improve efficiency, services, or innovation. BPMS and RPA are expected to improve efficiency. Cloud and the internet are focused on improving services, and AI and blockchain are expected to innovate and transform SSC. However, blockchain is expected to be less impactful than AI.

AI/ML emerged as the technology that should mostly impact the transformation of SSCs, providing a high level of innovation. AI/ML is followed by business analytics/data analytics, whose impact is expected to be high but directed towards improving services.

About AI, panelist P24, an SSC expert, said:

“Artificial Intelligence systems to i) provide detailed analytics to enable further process optimization - e.g., what's always 100% approved and therefore no longer requires an approval step, but more importantly; ii) prediction tools to spot trends and therefore proactively source solutions for internal or external clients; iii) 'holistic patterning' moving away from traditional silo workflows to spot subtle interrelationships and then built up process workflows that are more complex but provide greater client value and cost reduction.”

About analytics, P55, an SSC director, commented:

“Analytics will emerge as a significant game-changer. Shared Services are privy to a plethora of data. The ways this data can be converted to valuable information and then to insights are many. That is where the industry is headed.”

### Skills Required from SSC Professionals in 2030

Regarding the primary employee skills demanded by SSCs within 10 years, the top two were critical thinking (72%) and collaboration (59%). The technical knowledge of expertise area and problem-solving skills were ranked in third place, both having 50%. On critical thinking, P55, an SSC director, expressed:

“Humans will remain relevant because of critical thinking. That is something which you cannot impart on the hyper-automation solutions. To achieve that, the base has to be in the domain knowledge and technical expertise as well as a focus on the big picture. Collaboration with different moving parts and adaptation to change will become critical in determining success.”

As for the primary skills of leaders, they were found to be relevant: innovation and creativity (91%), new business opportunities (78%), high-performance team management (78%), new models of work organization (66%), and strategic management (59%). Panelist P59, an SSC director, said the following about creativity:

“The new leaders will need a strong knowledge in innovation, technology, and creativity to find new business opportunities to improve efficiency, efficacy, and users experience in the business processes.”

### SSC Business Models Transformation

The essence of the SSC concept resides in its business model, which “tries to capture the benefits of both centralization and decentralization” (Janssen & Joha, 2006b). Therefore, it is essential to understand how new technologies may transform the business model. Based on Díaz, Muñoz, and González (Díaz-Díaz et al., 2017), for our study, we defined business model as how the SSC creates and delivers value to customers and how the revenue is generated.

In the opinion of 66% of the panelists, the service scope will expand to a more analytical/high-end portfolio of services. Panelist P50, a shared services advisor, describes it this way:

“The SSC business model will change from FTE-centric to value delivered. People at SSCs will be engaged in doing high-end work and hence will be billed at a higher rate.”

In the words of panelist P32 (an SSC director):

“The SSC has to be more strategic in nature, providing not only timely, accurate, controlled information, but also entering into a business partnership relationship with customers, being responsible for managing the end-to-end process activities. The additional analytics provision by the SSC has the power to provide a competitive advantage to the business, in the marketplace.”

In the opinion of most panelists in the first Delphi round (62%), the current SSCs add value, mainly by offering cost reduction to the organization. Considering the 10-year horizon, they consider that the primary added value will become a more strategic action of the SSCs (44%). As panelist P29, an SSC researcher, said:

“It might go from either a cost center or specific center of excellence based on human labor (and its limitations) towards a value center whereby the underlying integrated and automated technology combines the best of both worlds by reducing costs and improving the service quality and user experience using AI. As such, it could become the engine of strategic innovation, and have a fundamental role in achieving and gaining competitive advantage for an organization.”

Most panelists (65%) understand that the need for SSCs will increase and that they will play a much more strategic role.

## Discussion of the Impact on SSCs

The study made it clear that while AI is expected to be the most influential technology, all six highlighted technologies can lead to the SSC reinvention, in all organizational dimensions. In the following, we discuss the overall impact of technology on SSCs, as well as in the profile of SSC employees and their business model.

### Impact of New Technologies by 2030

**Fig. 2:**
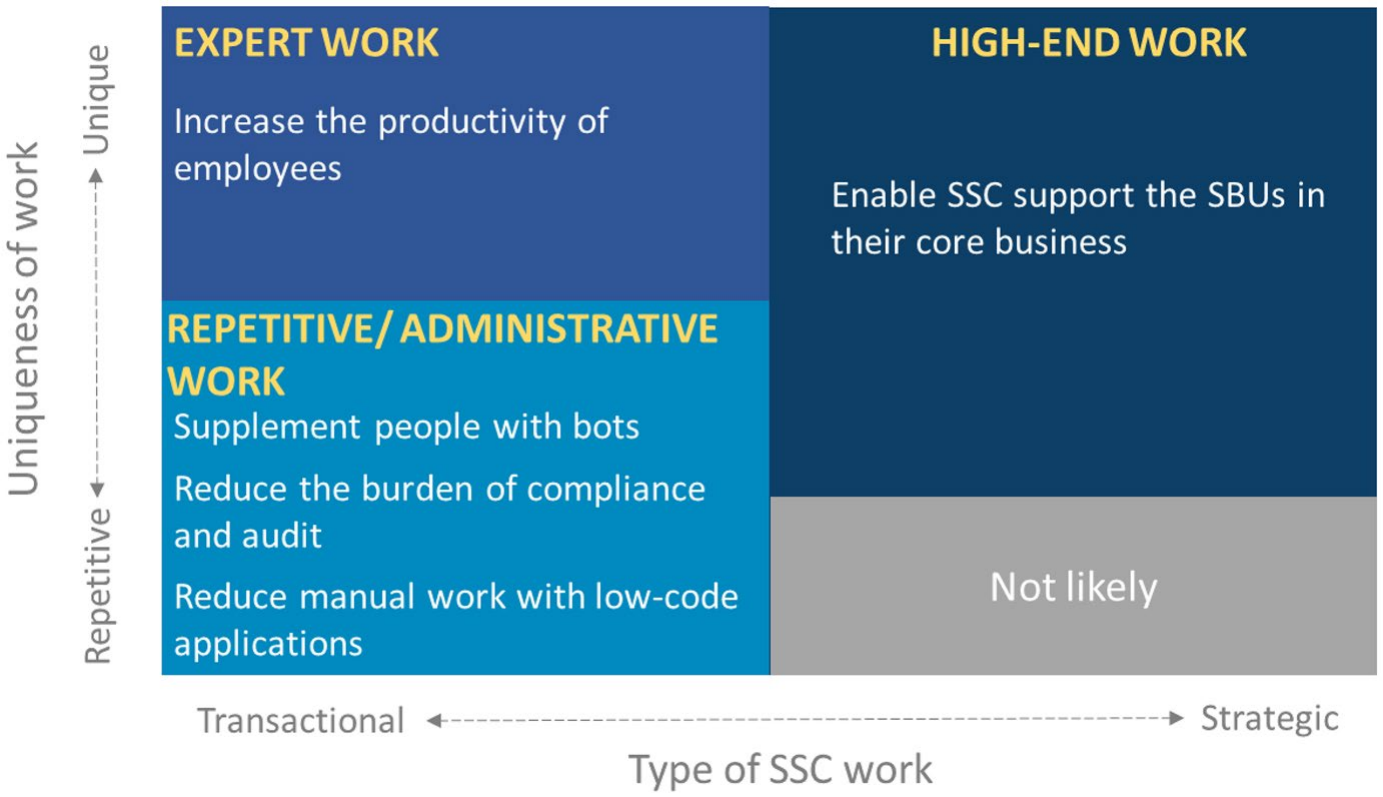
The emerging technologies disrupting the SSC work

The Delphi panelists anticipate a better and more intense use of technology by SSCs. In 2020, SSCs use technology primarily to automate repetitive work, adopting solutions such as basic RPA, ERPs, and industry-specific packages (e.g., core banking, insurance, or HR). In this way, technology supports the role of SSCs in reducing operating costs and cycles and improving data accuracy and service quality. However, by 2030, the technology will be used in a much bolder way. Going far beyond the automation of repetitive operational work, the technology will increase employee productivity in expert work, as Fig. 2 shows us. This trend was also found in Deloitte consultancy’s survey, with 379 respondents, from several countries, across nine industries (Deloitte, 2019). Contingency theory explains that for an organization to remain competitive, it is necessary to stay updated and incorporate the technology available in its context (Donaldson, 2001). It also explains that SSCs will adopt different strategies to meet their needs as organizations are all different. The technological transformation foreseen by the panelists will result in SSCs transformation to make them more competitive. Consequently, technology will no longer only contribute to cost reduction. Still, it will enable the reinvention of SSCs, which will provide higher added value services for organizations as a whole—repetitive work, administrative work, expert work, and high-end work (which will contribute to the SBUs’ core activities).

Currently, the services provided by SSCs are predominantly transactional, mostly record-keeping, administrative, and information provision tasks, with short turnaround (e.g., payroll, accounting, accounts payable, account receivable). These transactional tasks usually occur in a business process and can be easily automated with standard technology (e.g., ERP, workflow, basic RPA). The study identified that by 2030, these activities are likely to have been completely standardized and automated and that a digital worker/robot workforce will perform them. With the support of technologies such as AI and predictive modeling, they will be able to provide more strategic services by aggregating the support to revenue generation to the traditional cost reduction. The strategic work could include support for innovative product development, business decision-making, collaboration, and negotiation. SSCs are expected to play an active role in achieving and gaining a competitive advantage for an organization.

In the next two subsections, we discuss what changes will likely occur in the profile of professionals working in SSCs by 2030 and how the business model will be impacted.

### Employees’ and Managers’ Profile

**Fig. 3:**
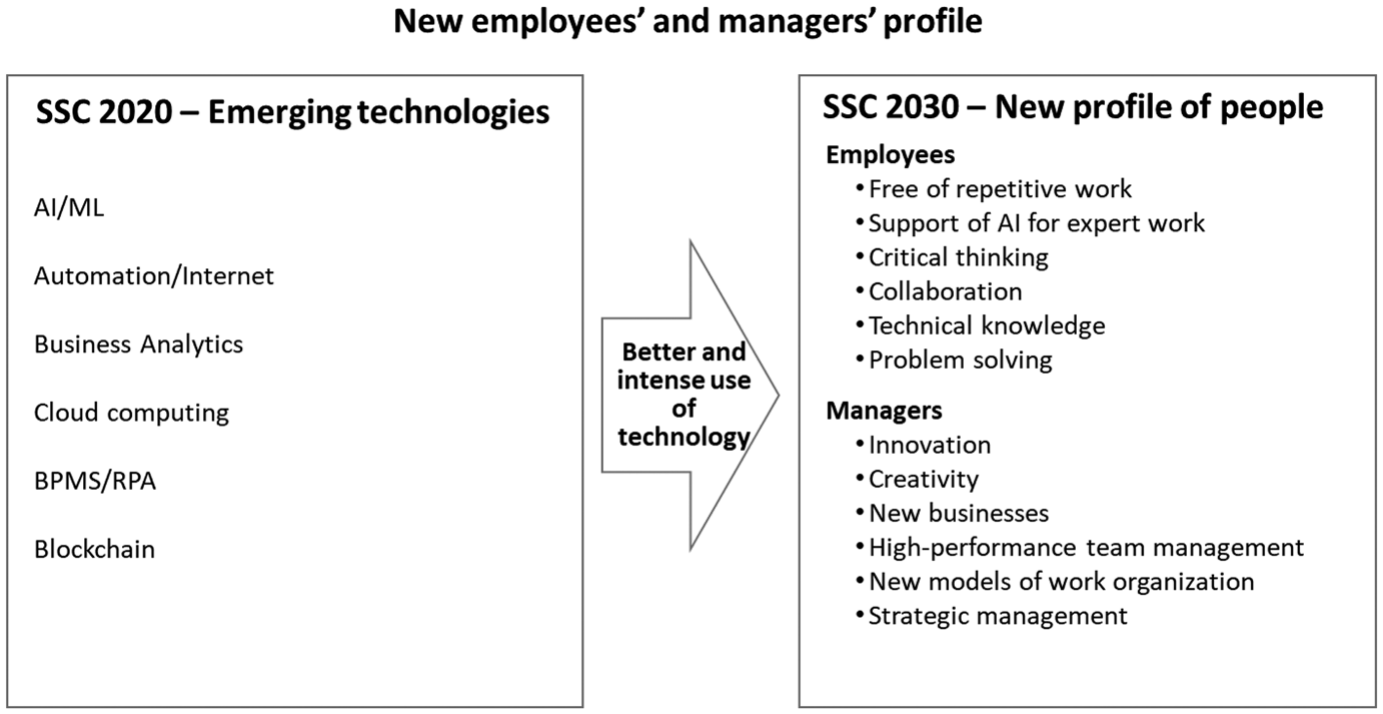
New employees’ and managers’ profile

As represented in Fig. 3 below, technology will free human beings from repetitive work and support them in more expert work; SSCs will likely need proportionally fewer and more skilled professionals. The skills of SSCs employees will change significantly. Employees’ critical thinking and collaboration will be essential skills in the future. Still, technical knowledge of the expertise area and problem-solving skills will also be relevant. Innovation and creativity, new business opportunities, and high-performance team management will be the most vital skills desired for managers. However, mastering new models of work organization—including digital workforce management—and strategic management will also be vital for them.

Dynamic capabilities theory helps clarify the dimensions that technology will impact, such as the organizational design and the execution environment of tasks, leading to the change of SSC capabilities—e.g., competencies, processes, learning, adaptability, decision-making, and organizational skills. The working environment of SSCs will undergo major organizational and work process transformations, resulting from the need for robotic and human workforce management and BPMS/RPA automated processes, and perhaps more importantly, from AI/ML applied to commercially relevant discoveries. Another aspect to consider is that these new professional profiles are more easily found in more developed countries, where labor costs are higher. Other research has already verified a growing migration of SSCs to countries with high labor costs (Deloitte, 2019; PwC, 2019).

### Business Model

On the business model of the SSCs, as defined in Sect. 1, the “Introduction,” and Subsection 4.3, the “SSC Business Models Transformation,” there will be a significant impact of the technology emerging today. Current operational challenges, such as manual and repetitive tasks, are expected to be resolved at that time because BPMS/RPA will have addressed them. The focus of the services provided by them should migrate from operational efficiency to higher value-added services. The latter is supported by cloud computing and internet and innovation support services, with AI/ML, blockchain, and business analytics. SSC’s scope of services will expand to a more analytical/high-end portfolio of services. As we saw in the research results, it is expected that the need for SSCs will increase (93% of the respondents), but its services will be much more strategic (87%). Open systems theory explains this transformation of the business model because the survival of SSCs depends on their ability to respond to the external environment, e.g., responding to the technological evolution and the risk of substitution by other equivalent service providers. Also, from the ST, we understand that the change of SSCs will provoke other organizational changes, for example, in the business units, which may delegate activities of greater added value to SSCs. Recent PwC research, with about 160 SSCs worldwide, corroborates the trend of changing the service portfolio of SSCs that increasingly move from transactional to knowledge-based processes (PwC, 2019). This trend was also confirmed by a recent Deloitte survey (Deloitte, 2019).

## Conclusions

The goal of this paper is to better understand technologies’ impact on SSCs in 2030 by conducting an empirical study. In this way, this research explored the dynamics of the knowledge economy and balances theory and practice. Existing technology plays a role in helping SSCs in their mission to reduce operating costs. In contrast, experts expect a disruption to happen. Six technologies are expected to significantly impact SSCs by 2030: AI/ML, packaged-based automation, BPMS/RPA, business analytics, blockchain, and cloud computing. These technologies will contribute to improved efficiency, new and better services, and innovations. AI technology is expected to have the highest impact and is most associated with innovation by the surveyed experts. Blockchain will also be associated with innovation services, but with less relevance than AI. BPMS/RPA will contribute to improving process efficiency. Business analytics/data analytics and internet and cloud computing will have a high impact on services.

The results suggest that the experts think significant transformations will happen at an intense pace. The new technologies will consolidate the digital transformation of SSCs and are expected to cause disruptions and accelerate the change of SSCs, impacting the way they operate and do business. The theoretical implication is that more research in SSC transformation is required, which functions are necessary, and how readiness for transformation can be measured and developed.

The study suggests that significant transformations are needed in two organizational dimensions: professional skills and the SSC business model. The profile of SSC employees is increasingly moving from being the executors of transactional activities to knowledge workers, who are involved in more complex activities operating at the more strategic level and being highly specialized. For skills, as the work’s nature will change to more complex and strategic work, it will be required from employees’ critical thinking and collaboration and from managers to drive innovation and creativity, new business opportunities, and high-performance team management. Hence, researchers should give more attention to the role of human resources in SSC and investigate which competencies and capabilities are needed to be future-proof. Perhaps, this study’s main contribution is the need for transformation in the business model. According to the experts, the transformation will happen to let the SSCs in becoming a strategic partner of the business units by adding value directly to their core business. This implies the need to theorize the business model dimensions of SSC better.

This study brings the following theoretical, policy, technical, and managerial implications. In theoretical terms, by exploring the future of SSCs, the study broadens the understanding of the relevance of SSCs for organizations and offers likely scenarios for their evolution, paving the way for further research in this field. The study suggests that there will be a reinvention of the SSC concept, as SSCs will no longer be focused on cost reduction and transactional activities, but will instead contribute directly to the core business, and the new technologies will support this trend. In policy terms, perhaps the main implication is that now policymakers will be able to anticipate the changes identified by this paper, preparing organizations and SSCs to have a combined transformation strategy and get the most out of the new SSC. In technical terms, the more intense use of the technologies addressed by the study will cause an increase in the complexity of the technological environment, which needs to be developed and managed. Specifically, security and maintenance costs should be given attention. Another implication is the transformation of the technical profile of SSCs’ employees, as they will contribute in a much more strategic way, going beyond the organization’s boundaries and considering closely their clients, products, competitors, among others. In managerial terms, the implications are remarkable, as SSC managers will need to be visionary leaders, able to see both the forest and the trees, and lead from digital workforces to high-skilled knowledge workers and self-managed SSC teams, among others. In addition, they should lead the transition from today’s SSCs to the SSCs of the future, with all the inherent challenges. Perhaps, one of the major challenges is preparing for this future from this day forward and beginning the change now.

Although the study’s learning has limited generalizability, managers responsible for SSCs can benefit and plan the adaptation of their SSCs to the foreseen changes presented in this article. SSCs will likely change, but there might be many influences and endogenous developments that might influence how this will occur.

Recommendations for new research could include verifying how the Covid-19 pandemic anticipated or accelerated the digital transformation of SSCs and, consequently, using the technologies covered in this study. Another research problem could involve studying professionals’ transition today dedicated to transactional work to more complex, strategic works. Could it be the same people, trained for the new functions, or mandatorily new professionals with profiles so different that they make it impossible to leverage the current professionals? Finally, systems theory shows us that we need to understand the entire organization, not just one of its parts. Thus, our suggestion for future studies is to confirm the results of this research from the perspective of the internal clients of the SSCs. Our study focused on SSC executives and specialists, and the new studies could be based on the view of SSC clients. What do they expect and anticipate?

## Appendix

### Appendix 1. First questionnaire

Link: https://drive.google.com/file/d/1ORNgUxEnl9WrtnhysFIG9OSRlgfPQeZ3/view?usp=sharing

### Appendix 2. Second questionnaire

Link: https://drive.google.com/file/d/16n6UtuLSCaIt_m9PLPoxoX4JSRVCeL-Y/view?usp=sharing

## Declarations

### Potential Reviewers in the Field of Shared Services and Transformation

Anton Joha, email: anton.joha@smylyx.com; Vishanth Weerakkody, email: V.Weerakkody@bradford.ac.uk.
